# Rare huge retroperitoneal cystic lymphangioma presenting as acute abdomen in an adult

**DOI:** 10.1259/bjrcr.20170120

**Published:** 2018-04-05

**Authors:** Iyiade Olatunde Olaoye, Micheal Dapo Adesina

**Affiliations:** Surgery Department, University of Ilorin Teaching Hospital Ilorin, Ilorin, Nigeria

## Abstract

Cystic lymphangiomas are rare benign tumors. Most are diagnosed in childhood and their presentation in adults is rare. Retroperitoneal cystic lymphangiomas constitute only 1% of lymphangiomas. Unfortunately the differentiation between cystic lymphangiomas and other cystic tumors is often not possible and surgery with histology is essential for confirmation of diagnosis. A 20-year-old lady with retroperitoneal cystic lymphangioma presented with acute abdomen. In the diagnosis of this patient, abdominal Ultrasound, CT and MRI scans were obtained.

## INTRODUCTION

Cystic lymphangiomas are rare benign tumors of the lymphatic system. More than 90% are diagnosed in childhood. They are rarely diagnosed in adults. Only 1% of cystic lymphangiomas are retroperitoneal. The clinical presentation of retroperitoneal cystic lymphangioma varies widely and radiological imaging is very important although the definitive diagnosis is from histology and immunohistochemistry. A 20-year-old lady presented with acute severe epigastric pain and a tender epigastric mass. Ultrasound, CT and MRI images surprisingly revealed features of retroperitoneal cystic lymphangioma.

## CASE REPORT

**Figure 1.**
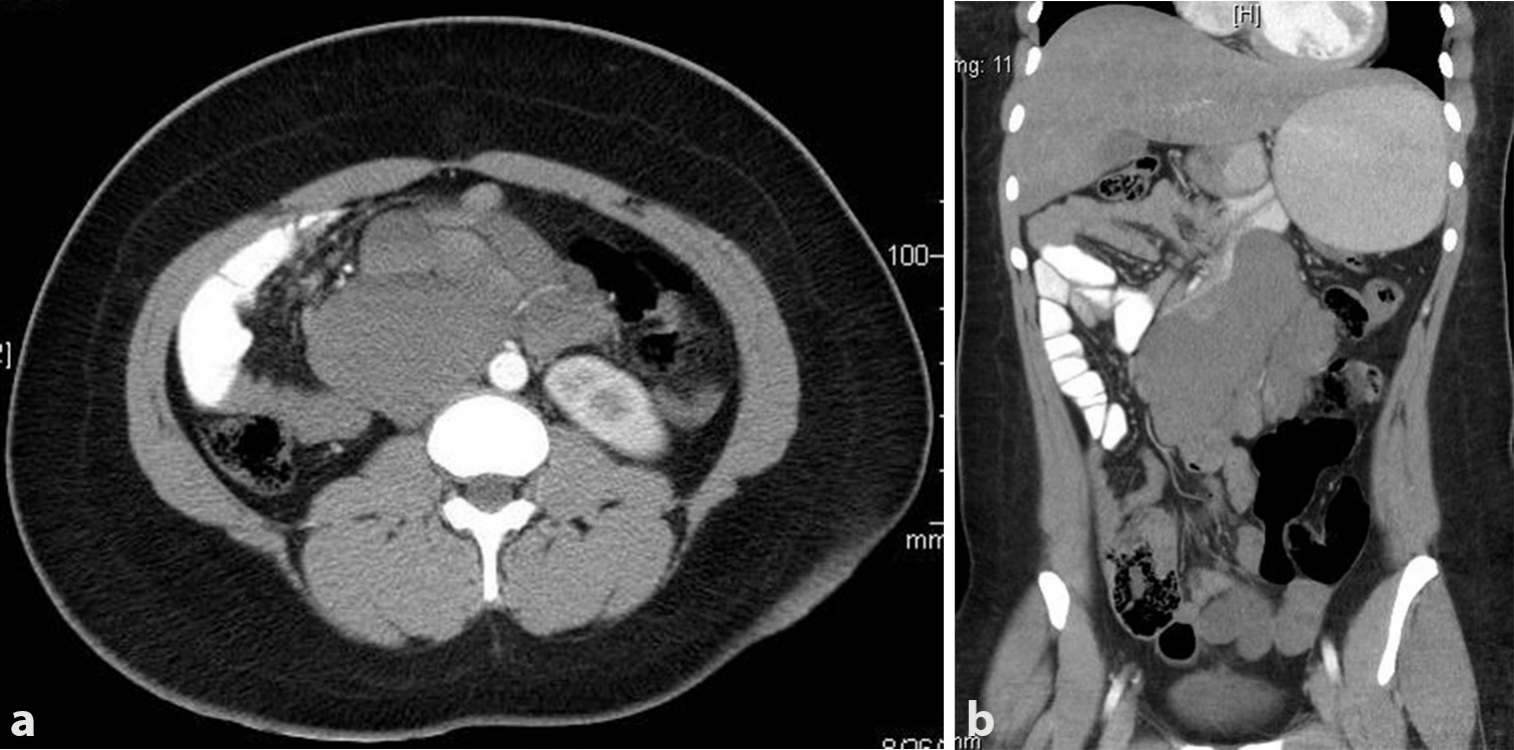
(a) Contrast axial abdominal CT with a retroperitoneal multiseptated homogeneous cyst. (b) Contrast abdominal CT coronal view with a retroperitoneal multiseptated homogeneous cyst occupying more than one compartment.

**Figure 2.**
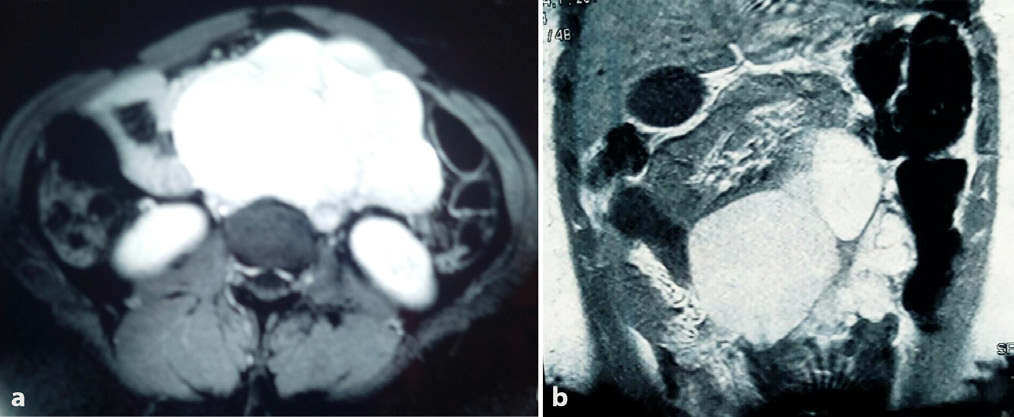
(a) Axial abdominal MRI showing retroperitoneal multiple well defined round uniformly dense cystic masses. (b) Abdominal MRI coronal view showing retroperitoneal multiple well-defined round uniformly dense cystic masses occupying more than a compartment.

A 20-year-old lady presented with sudden severe non-colicky upper abdominal pain. She had no history of trauma to the abdomen and no previous episodes. Her appetite and bowel habits had been normal. Examination revealed an otherwise healthy young lady in painful distress and with a tender firm epigastric mass that extended from the rib cage down to the level of the umbilicus. It felt smooth and fixed. She was not pale and was hemodynamically stable. Abdominal ultrasonography showed a multiloculated cyst in the upper abdomen extending over the pancreas and suggested a pancreatic pseudocyst. Her pain got better on analgesics. An abdominal CT obtained a week after the initial presentation (Figure 1a,b) revealed a multiseptated cystic retroperitoneal mass with homogenous fluid located below the liver, extending downwards over the pancreas and the right kidney and over the medial edge of the left kidney. It pushed the stomach superiorly and extended to reach the anterior abdominal wall. There was no evidence of acute hemorrhage into the cyst. MRI (Figure 2a,b) showed a multicystic uniformly dense mass with long signal intensity that extended just below the anterior abdominal wall and suggested a mesenteric cyst. It was separate from major organs. Features of acute hemorrhage were also absent.

**Figure 3.**
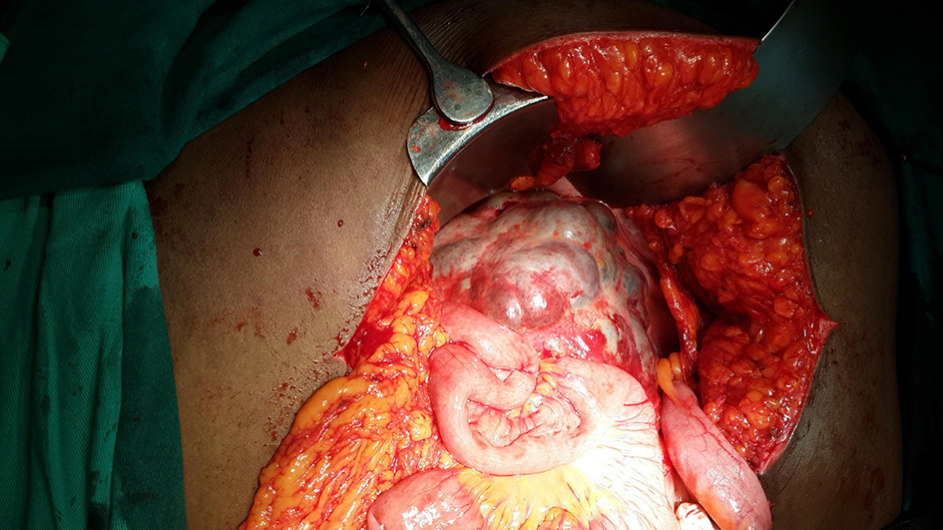
Intraoperative findings including a retroperitoneal multicystic mass around the proximal jejunum.

Exploratory laparotomy revealed a multicystic mass with thin walls in the retroperitoneum located below the liver (Figure 3). It extended over the pancreas but was not attached to it. It surrounded the retroperitoneal part of the proximal jejunum. The stomach and transverse colon were displaced superiorly and the small intestine inferiorly. Some of the cysts contained brownish fluid from altered blood. The mass was completely excised without bowel resection. Histology confirmed a cystic lymphangioma. She had an unremarkable post-operative period and has been followed up for 3 years without recurrence.

## DISCUSSION

First described by Koch in 1913, cystic lymphangiomas are widely accepted to be congenital malformations from failure of channelization and drainage of lymphatic sacs with resultant dilatation of the involved lymphatic vessels.^1, 2^ Some cases may result from trauma, surgery, infection and irradiation.^3^ Most present in childhood and are more common in males. They are rarely discovered in adults. 75% of cystic lymphangiomas are located in the neck, 20% in the axilla. About 5% are located in the abdomen in the mesentery of the small and large bowel, the omentum, and less commonly the liver, spleen, kidney and pancreas. 1% of cystic lymphangiomas are located in the retroperitoneum. The presentation of retroperitoneal cystic lymphangioma is not specific and pre-operative diagnosis depends very much on radiological imaging.^4^ Most are asymptomatic incidental findings during radiological investigation of the abdomen, at laparotomy, laparoscopy, or at autopsy. They may present with chronic abdominal pain, chronic back pain or acutely with intestinal obstruction, ureteric obstruction, sepsis, torsion and disseminated intravascular coagulation.^5^ Our patient presented with acute abdominal pain from hemorrhage into the cyst.

Diagnosis and approach to management of cystic retroperitoneal lesions can be highly challenging and pre- operative diagnosis complex.^6^ Rare presentation of retroperitoneal lymphangioma in adults may make radiological diagnosis even more challenging. Ultrasonography, CT, MRI and laparoscopy are useful in diagnosis although the definitive diagnosis requires histology and an immunohistochemistry.^7^ The differential diagnosis of cystic retroperitoneal lesions include cystic mesothelioma, teratoma, undifferentiated sarcoma, cystic metastasis, malignant mesenchymoma, cyst of urethelial or foregut origin, microcystic pancreatic adenoma, pancreatic pseudocyst, ovarian cyst, duplication cyst, retroperitoneal hematoma and abscesses.^8^ Sonographically, lymphangiomas are most often multilocular cystic masses that are anechoic or contain echogenic debris. In contrast-enhanced CT, the fluid component is typically homogeneous with low attenuation values but occasionally, negative attenuation values occur in the presence of chyle. The lesion may occupy more than one retroperitoneal compartment.^9, 10^ Calcification is uncommon. The MRI finding is that of well-defined round or oval uniformly dense cystic masses with long signal intensity on *T*_1_ and *T*_2_ weighted images.^6^ The ability of MRI to provide images in multiple planes with no loss of resolution may demonstrate additional lesions and further delineate the boundaries of the cysts.^11^ MRI allows a detailed assessment of the lesions morphology and structure, shows vessel-like internal septa, wall thickness and fluid content better. It also excludes the presence of mucoid, adipose or solid component.^12^ Definitive diagnosis of cystic lymphangioma requires histology and immunohistochemistry. At histology, cystic lymphangiomas are classified into capillary, cavernous and cystic types. Nearly all retroperitoneal lymphangiomas are cystic. Immunohistochemistry of cystic lymphangioma shows endothelial cells that express factor VIII-related antigen, CD31 and CD34.^13^

Laparotomy and complete excision is the treatment of symptomatic retroperitoneal cystic lymphangioma. Excision is required for diagnosis in asymptomatic cases and to prevent complications. Laparoscopic excision of a similarly huge retroperitoneal cystic lymphangioma after catheter aspiration of the fluid has been documented.^14^

## LEARNING POINTS

1. Retroperitoneal cystic lymphangioma is a rare benign tumor in adults with unspecific presentation. It was a surprise finding in this case of acute abdomen.
2. At CT, cystic lymphangioma typically appears as a large, thin walled, multiseptate cystic mass. Diagnostic clue for cystic lymphangioma: an elongated shape and crossing from one retroperitoneal compartment to an adjacent one.
3. Because the presentation of retroperitoneal cystic lymphangioma is not specific and preoperative diagnosis coupled with approach to management highly dependent on radiological imaging, familiarity with the radiological images is essential to management.

## Informed consent

Written informed consent for the case to be published (including images, case history and data) was obtained from the patient(s) for publication of this case report, including accompanying images.

## References

[1] Koch K Beitrage Zur Patholgie der Bauchspeicheldruse. Virchows Achiv ffur Pathologische A natomie und Physioliogie und fur Klinische Medizin 1913; 214: 180–206.

[2] Nuzzo G, Lemmo G, Marrocco-Trischitta MM, Boldrini G, Giovannini I Retroperitoneal cystic lymphangioma. J Surg Oncol 1996; 61: 234–7. doi: 10.1002/(SICI)1096-9098(199603)61:3&lt;234::AID-JSO14&gt;3.0.CO;2-7 8637214

[3] Allen JG, Riall TS, Cameron JL, Askin FB, Hruban RH, Campbell KA Abdominal lymphangiomas in adults. J Gastrointest Surg 2006; 10: 746–51. doi: 10.1016/j.gassur.2005.10.015 16713549

[4] Ge W, Yu DC, Chen J, Shi XB, Su L, Ye Q, et al Lymphocele: a clinical analysis of 19 cases. Int J Clin Exp Med 2015; 8: 7342–50. 26221274 PMC4509219

[5] Gachabayov M, Kubachev K, Abdullaev E, Babyshin V, Neronov D, Abdullaev A A Huge Cystic Retroperitoneal Lymphangioma Presenting with Back Pain. Case Rep Med 2016; 2016: 1–4. doi: 10.1155/2016/1618393 PMC5097799 27843456

[6] Morotti A, Busso M, Consiglio Barozzino M, Cinardo P, Angelino V, Familiari U, Barozzino MC, et al Detection and management of retroperitoneal cystic lesions: a case report and review of the literature. Oncol Lett 2017; 14: 1602–8. doi: 10.3892/ol.2017.6323 28789385 PMC5529955

[7] Saadi A, Ayed H, Karray O, Kerkeni W, Bouzouita A, Cherif M, et al Retroperitoneal cystic lymphangioma: about 5 cases and review of the literature. Pan Afr Med J 2016; 25: 73. doi: 10.11604/pamj.2016.25.73.10002 28292036 PMC5324145

[8] Bonhomme A, Broeders A, Oyen RH, Stas M, De Wever I, Baert AL Cystic lymphangioma of the retroperitoneum. Clin Radiol 2001; 56: 156–8. doi: 10.1053/crad.2000.0162 11222077

[9] Levy AD, Cantisani V, Miettinen M Abdominal lymphangiomas: imaging features with pathologic correlation. AJR Am J Roentgenol 2004; 182: 1485–91. doi: 10.2214/ajr.182.6.1821485 15149994

[10] Yang DM, Jung DH, Kim H, Kang JH, Kim SH, Kim JH, et al Retroperitoneal cystic masses: CT, clinical, and pathologic findings and literature review. Radiographics 2004; 24: 1353–65. doi: 10.1148/rg.245045017 15371613

[11] Cutillo DP, Swayne LC, Cucco J, Dougan H CT and MR imaging in cystic abdominal lymphangiomatosis. J Comput Assist Tomogr 1989; 13: 534–6. doi: 10.1097/00004728-198905000-00038 2656785

[12] Romeo V, Maurea S, Mainenti PP, Camera L, Aprea G, Cozzolino I, et al Correlative imaging of cystic lymphangiomas: ultrasound, CT and MRI comparison. Acta Radiol Open 2015; 4: 204798161456491 May. doi: 10.1177/2047981614564911 PMC4437906 26019889

[13] DI Marco M, Grassi E, Vecchiarelli S, Durante S, Macchini M, Biasco G Retroperitoneal lymphangioma: a report of 2 cases and a review of the literature regarding the differential diagnoses of retroperitoneal cystic masses. Oncol Lett 2016; 11: 3161–6. doi: 10.3892/ol.2016.4367 27123082 PMC4841079

[14] Ishibashi Y, Tsujimoto H, Kouzu K, Horiguchi H, Nomura S, Ito N, et al Laparoscopic resection of a huge retroperitoneal cystic lymphangioma after successful reduction of tumor size with a double balloon catheter. Int J Surg Case Rep 2015; 11: 8–10. doi: 10.1016/j.ijscr.2015.04.016 25898335 PMC4446689

